# Combined Multi-Omics Analysis Reveals the Potential Role of *ACADS* in Yak Intramuscular Fat Deposition

**DOI:** 10.3390/ijms25169131

**Published:** 2024-08-22

**Authors:** Fang Xu, Haibo Wang, Chunyu Qin, Binglin Yue, Youzhualamu Yang, Jikun Wang, Jincheng Zhong, Hui Wang

**Affiliations:** Key Laboratory of Qinghai-Tibetan Plateau Animal Genetic Resource Reservation and Utilization, Sichuan Province and Ministry of Education, Southwest Minzu University, Chengdu 610225, China; xufang1172@163.com (F.X.); wanghaibo@swun.edu.cn (H.W.); 220710002005@stu.swun.edu.cn (C.Q.); yuebinglin123@163.com (B.Y.); yangyouzlm@163.com (Y.Y.); xdzwjk@163.com (J.W.); zhongjincheng518@126.com (J.Z.)

**Keywords:** transcriptome, proteome, *ACADS*, IMF, PPARα, yak

## Abstract

The Yak (*Bos grunniens*) is a special breed of livestock predominantly distributed in the Qinghai–Tibet Plateau of China. Intramuscular fat (IMF) content in beef cattle is a vital indicator of meat quality. In this study, RNA-Seq and Protein-Seq were respectively employed to sequence the transcriptome and proteome of the *longissimus dorsi* (LD) tissue from 4-year-old yaks with significant differences in IMF content under the same fattening conditions. Five overlapping genes (*MYL3*, *ACADS*, *L2HGDH*, *IGFN1*, and *ENSBGRG00000000-926*) were screened using combined analysis. Functional verification tests demonstrated that the key gene *ACADS* inhibited yak intramuscular preadipocyte (YIMA) differentiation and proliferation, promoted mitochondrial biogenesis gene expression, and increased the mitochondrial membrane potential (MMP). Furthermore, co-transfection experiments further demonstrated that interfering with *ACADS* reversed the effect of PPARα agonists in promoting lipid differentiation. In conclusion, *ACADS* potentially inhibits lipid deposition in YIAMs by regulating the PPARα signalling pathway. These findings offer insights into the molecular mechanisms underlying yak meat quality.

## 1. Introduction

Yaks, primarily distributed in the Qinghai–Tibet Plateau, is an important characteristic genetic resource in China. They exhibit remarkable adaptability to high altitudes, extremely cold temperatures, and intense ultraviolet radiation and play a crucial role in providing production and livelihood materials for farmers and herders on the Qinghai–Tibet Plateau [1]. Yak meat is popular because of its rich protein levels, low cholesterol content, and abundant minerals [2]. However, yaks have slower growth, muscle development, and fat deposition efficiency than ordinary cattle of the same age. Consequently, yak meat tends to be tougher and has a rough taste, which decreases its economic benefits within the Qinghai–Tibet Plateau [3]. Therefore, enhancing muscle development and fat deposition in yak meat is one method of improving the tenderness and taste. This is also important for promoting the sustainable development of the yak breeding industry and enhancing its economic benefits [4].

Mammalian adipose tissues can be categorised into three types: subcutaneous, intramuscular, and visceral. Intramuscular fat (IMF) belongs to the fat in skeletal muscle, including adipose tissue between muscle fibres and lipid droplets in muscle cells [5]. The IMF content is significantly correlated with the colour, tenderness, water retention, flavour, and juiciness of meat [6], and it represents an important indicator of meat quality because an appropriate amount of IMF enhances the taste and flavour of meat. IMF has a more pronounced impact on meat quality than excessive external fat [7] and represents a source of essential nutrients, such as conjugated linoleic acid and monounsaturated fatty acids, which offer certain benefits in reducing the incidence of cardiovascular disease and preventing atherosclerosis [8].

Genetic and environmental factors significantly influence the IMF content, and genetic advances and nutritional regulation are effective methods for enhancing the IMF content in meat products. Changes in the IMF content mainly involve the proliferation, differentiation, and maturation of adipocytes [9]. The regulation of IMF deposition can be divided into two processes: lipid synthesis and lipolysis [10]. Research has demonstrated that enzymes involved in fatty acid synthesis and lipolysis, such as monoacylglycerol lipase (MAGL), hormone-sensitive lipase (HSL), adipose triglyceride lipase (ATGL), fatty acid-binding protein (FABP), and lipoprotein lipase (LPL), control IMF deposition [11]. Acyl-CoA dehydrogenase short chain (*ACADS*) belongs to the *ACADS* family of mitochondrial enzymes and is primarily involved in the first step of mitochondrial fatty acid β-oxidation [12]. The primary focus of current research regarding the *ACADS* family is the diagnosis, aetiology, and pathogenesis of acyl-CoA dehydrogenase deficiency. However, studies on the involvement of *ACADS* in lipid deposition and metabolism are still limited. Chen [13] determined the metabolism of *ACADS* knockout mice after high-fat diet feeding and found that these mice could resist obesity caused by high-fat diet feeding by hindering liver de novo lipogenesis (DNL). The liver of *ACADS* knockout mice fed low or high-fat diets were subjected to transcriptome sequencing, and the results showed that the PPARα signalling pathway and lipid processing pathway were significantly upregulated [12]. LC/MS-based analyses of glioma sphere-forming cells (GSCs) were conducted to investigate the impact of medium-chain acyl-CoA dehydrogenase (MCAD) deletion on cellular lipids and demonstrated that MCAD deletion significantly elevated the levels of triacylglycerol (TAG) and certain phospholipids [14]. These studies have shown that *ACADS* may be involved in the regulation of lipid metabolism.

At present, research on IMF deposition in yaks has mainly focused on using high-throughput sequencing technology to conduct differential gene screening and bioinformatic analyses at different energy levels or during different growth and development stages. However, research on key genes in the *longissimus dorsi* (LD) with different IMF contents under the same age and fattening conditions is lacking. Additionally, most research has been limited to genome and transcriptome analyses, which only provide a partial understanding of the changes involved in IMF deposition. Therefore, in this study, RNA-Seq and Proteome-Seq were respectively used to comprehensively analyse transcriptome and proteome data from the LD tissue of 4-year-old yaks with significant differences in IMF content under the same fattening conditions. We identified potential key genes that influence the IMF content in yaks by integrating omics data and investigated the functions and regulatory mechanisms of a key gene, *ACADS*.

## 2. Result

### 2.1. Overview of Transcriptome Data

**Figure 1:**
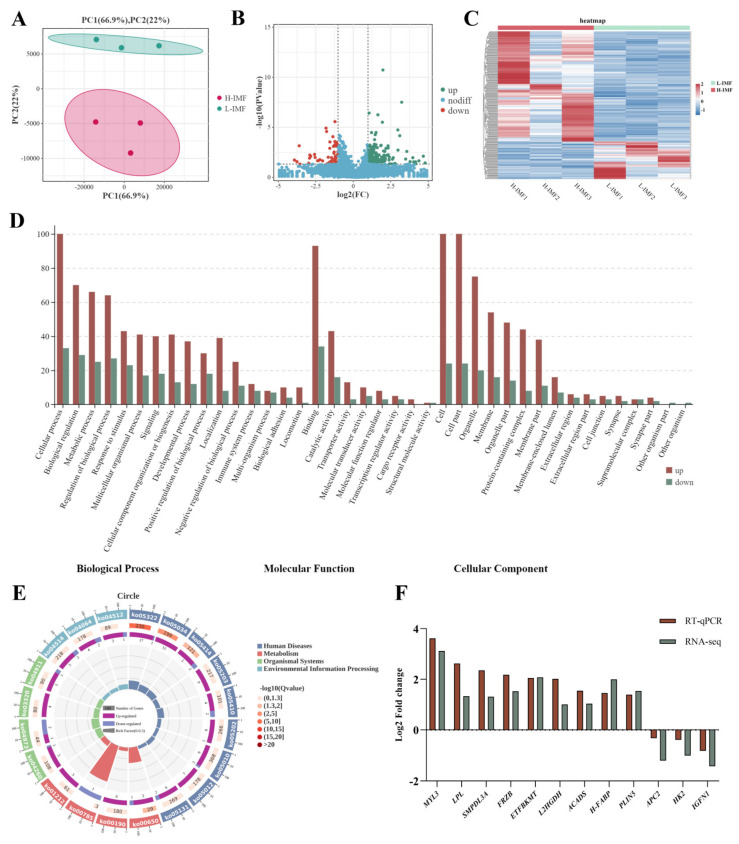
Overview of transcriptome data. (**A**) Principal component analysis (PCA). (**B**) Volcano plot of DEGs. (**C**) Heatmap of the hierarchical cluster analysis of all samples and DEGs. (**D**) Gene Ontology (GO) terms with significant enrichment of DEGs. (**E**) Circle map of the top 20 enriched KEGG pathways for DEGs. (**F**) RNA-Seq data verified by RT-qPCR.

The Illumina Novaseq6000 sequencing platform was used to sequence the cDNA library, and an average of 195,435,549 high-quality clean reads were obtained. The total alignment rate with the reference genome (Ensembl _ release 100) was 91.29% to 92.85% (Table S1). Principal component analysis (PCA) and Pearson’s correlation analysis revealed a difference between the lowest IMF content (L-IMF) and the highest IMF content (H-IMF) (Figure 1A). In addition, the R values of the three different repeated samples were greater than 0.98 (Figure S1A), indicating the good repeatability of the two groups of samples. After screening, a total of 256 differentially expressed genes (DEGs) (65 downregulated and 191 upregulated) were detected between the two groups (Figure 1B,C).

The DEGs were significantly enriched in 49 Gene Ontology (GO) terms (Figure 1D), including fatty acid beta-oxidation (GO: 0033539), positive regulation of lipid storage (GO: 1905954), positive regulation of triglyceride sequestration (GO: 0010890), and fatty acid metabolic processes (GO: 0006631) (Figure S1B), which are related to lipid formation and metabolism. Kyoto Encyclopaedia of Genes and Genomes (KEGG) pathway analysis showed that 256 DEGs were significantly enriched in 32 pathways, including the PPAR signalling pathway (ko03320), oxidative phosphorylation (ko00190), and regulation of fatty acid metabolism pathway (ko01212) (Figure 1E), which are related to fat growth and fat deposition.

Real-time quantitative polymerase chain reaction (RT-qPCR) verification was performed on 12 randomly selected DEGs (Figure 1F), including nine upregulated and three downregulated genes. This change in expression was consistent with the results of transcriptome sequencing, indicating that the expression profiles of the DEGs evaluated in this study were highly reliable.

### 2.2. Overview of Proteome Data

**Figure 2:**
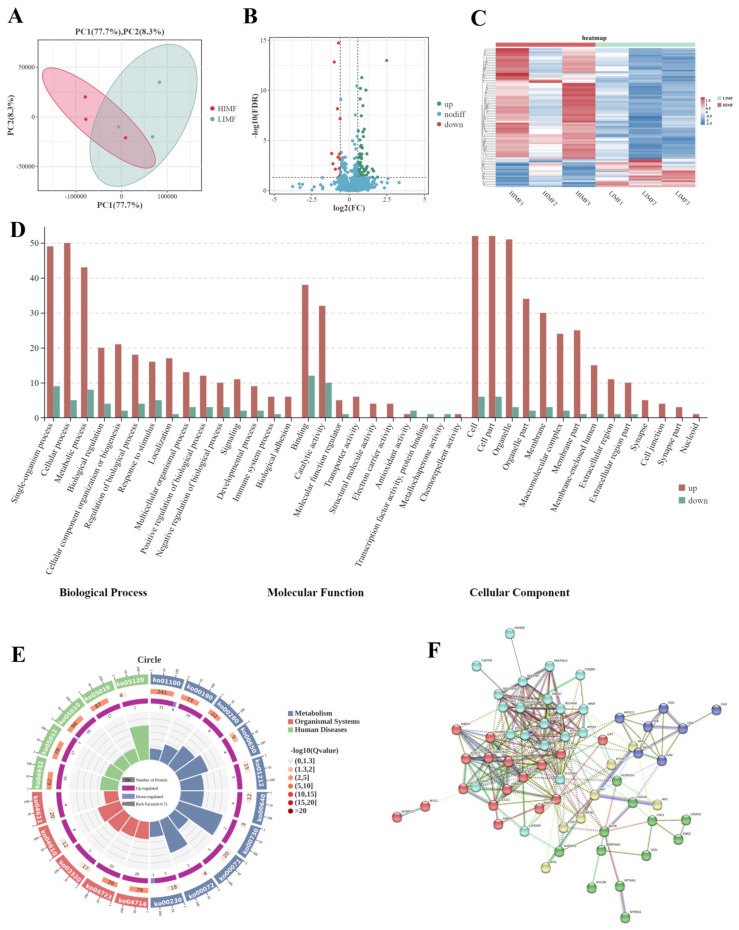
Overview of proteome data. (**A**) PCA for H-IMF and L-IMF. (**B**) Volcano plot for the distribution of DEPs. (**C**) Heatmap of the hierarchical cluster analysis of all sequencing samples and DEPs. (**D**) GO terms with significant enrichment of DEPs. (**E**) Circle map of the top 20 enriched KEGG pathways for DEPs. (**F**) Functional clustering of DEPs by STRING 12.0.

Proteome sequencing analysis revealed 8185 peptides, 1009 proteomes, and 1133 proteins (Figure S1C), most of which were composed of 1–10 peptides (Figure S1D). PCA and Pearson’s correlation analysis showed a difference between the L-IMF and H-IMF groups (Figure 2A). Furthermore, the R values of the three repeated samples were greater than 0.97 (Figure S1E), indicating good repeatability between the two groups. A total of 84 differentially expressed proteins (DEPs) (17 downregulated and 67 upregulated) were detected between the two groups (Figure 2B,C).

The GO functional enrichment analysis results showed that DEPs were significantly enriched in a total of 47 GO terms (Figure 2D), including lipid modification (GO: 0030258), fatty acid beta-oxidation (GO: 0006635), fatty acid catabolic processes (GO: 0009062), regulation of lipid transport (GO: 0032368), and cellular lipid metabolic processes (GO: 0044255) (Figure S1F). The KEGG pathway analysis results showed that the DEPs were significantly enriched in 18 pathways, including the PPAR signalling pathway (ko03320), fatty acid metabolism (ko01212), and fatty acid degradation (ko00071) (Figure 2E).

The results obtained from the protein-protein interaction (PPI) network analysis showed that all DEPs clustered into five groups (Figure 2F). The first group (light blue) was mainly involved in mitochondrial and energy metabolism, the second (red) was involved in fat synthesis and metabolism, the third (green) was involved in the cytoskeleton and cell junctions, the fourth (yellow) was involved in oxidative phosphorylation, and the fifth (dark blue) was involved in lipoprotein regulation.

### 2.3. Integrated Analysis of Transcriptome and Proteome Data

**Figure 3:**
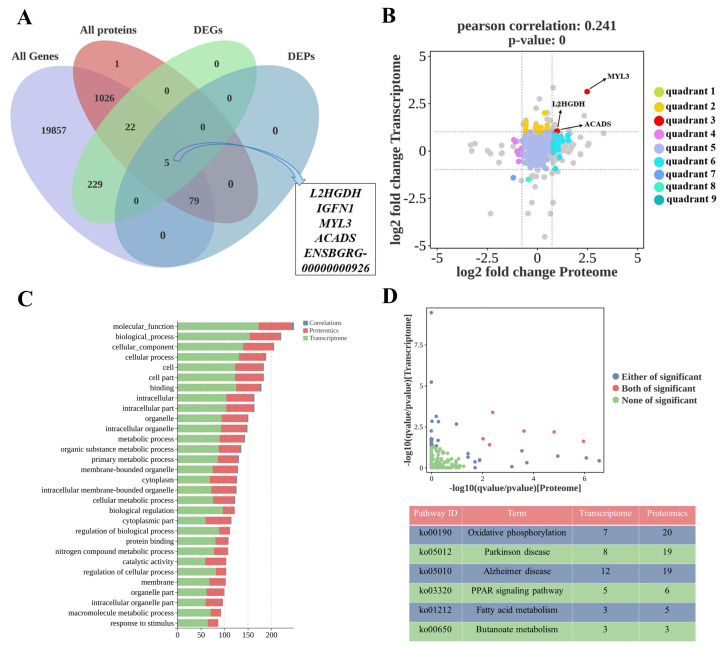
Combined analysis of transcriptome and proteome data. (**A**) Venn diagram of all genes, all proteins, DEGs and DEPs. (**B**) Nine-quadrant diagram of DEGs and DEPs. (**C**) Top 30 enriched GO terms for both transcriptome and proteome data. (**D**) Significantly enriched KEGG pathways for both transcriptomics and proteomics data.

Next, we performed an association analysis of the transcriptome and proteome data to more accurately screen for key genes and proteins that affect lipid deposition. Only five overlapping genes/proteins (Figure 3A) were shown on a Venn diagram: immunoglobulin-like and fibronectin type III domain containing 1 (IGFN1), myosin light chain3 (MYL3), *ACADS*, ENSBGRG00000000926, and L-2-hydroxyglutarate dehydrogenase (L2HGDH). The nine-quadrant map showed that the *MYL3*, *ACADS* and *L2HGDH* were mainly distributed in the third quadrant, and the expression patterns in the transcriptome and proteome were consistent, all of which were up-regulated (Figure 3B). The results of the combined analysis of GO terms showed that DEPs/DEGs with significant differences in both transcriptome and proteome were significantly enriched in molecular functions, biological processes, cellular components, cellular processes, intracellular parts, and biological regulation (Figure 3C). Moreover, the significantly enriched KEGG pathways for both omics data included the PPAR signalling pathway, Parkinson’s disease, fatty acid metabolism, Alzheimer’s disease, butanoate metabolism, and oxidative phosphorylation (Figure 3D).

### 2.4. ACADS Expression Profile Analysis

**Figure 4:**
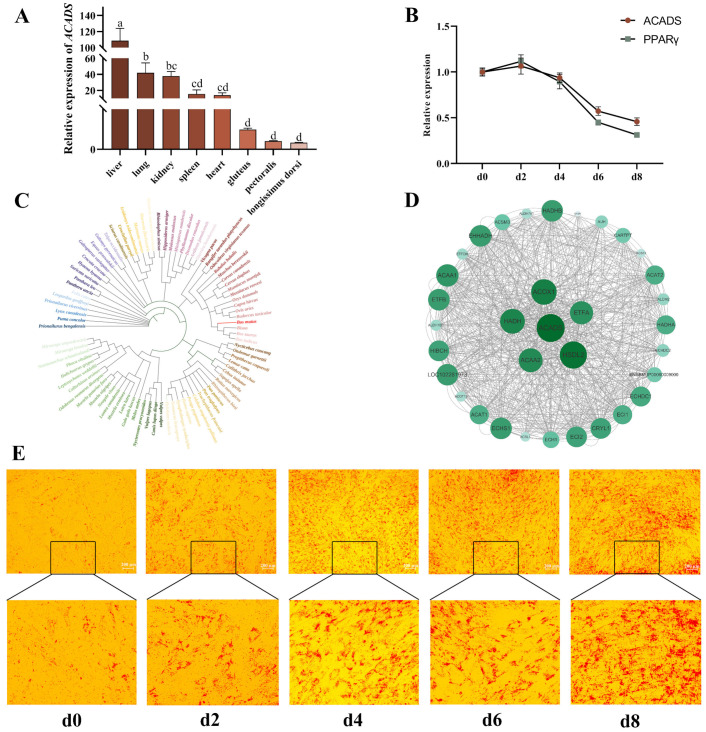
Analysis of the *ACADS* sequence and tissue expression profile. (**A**) Tissue expression pattern of *ACADS* in Maiwa yaks (n = 9), the same lowercase letter indicates that the difference is not significant (*p* > 0.05), and the different lowercase letters indicate significant differences (*p* < 0.05). (**B**) Expression levels of *ACADS* and *PPARγ* in YIMAs (n = 9). (**C**) Phylogenetic tree of *ACADS* protein sequence. (**D**) Protein interaction network of *ACADS*. (**E**) Construction of the lipid deposition model of YIMAs. Data are presented as the mean ± SEM.

Based on the results of the multi-omics combined analysis, we selected *ACADS* for subsequent functional verification due to the significant fold difference among the five overlapping genes. The tissue expression profile showed that the expression of *ACADS* in the liver of the yaks was much higher than that in the other tissues (Figure 4A). During adipogenic differentiation, the expression of the *ACADS* gene gradually decreased after reaching a peak on the second day. The expression of *PPARγ*, a marker gene of adipogenic differentiation, also increased and then decreased (Figure 4B). Sequence conservation analysis showed that the similarity of *ACADS* between yak and *Bos taurus* was the highest (99.2%), followed by that between yak and *buffalo* (98.39%) and *Qinling takin* (98.12%), while the similarity with *Homo sapiens* was 92.49% (Figure 4C). The PPI analysis results showed that proteins interacting with *ACADS* in yaks included ECHS1, ACAA1, ETFA, HADH and other key proteins related to fatty acid metabolism (Figure 4D). These results indicate that *ACADS* is a highly conserved protein mainly distributed in the liver and thus is related to fatty acid metabolism and may regulate the early differentiation of yak intramuscular preadipocytes (YIMAs). Next, oleic acid was used to induce cell differentiation in YIMAs (0–8 days) to explore the *ACADS* expression patterns during preadipocyte differentiation. The accumulation of lipid droplets gradually increased during differentiation (Figure 4E), indicating that the YIMA lipid differentiation model was successfully established.

### 2.5. ACADS Inhibits the Differentiation of YIMAs

**Figure 5:**
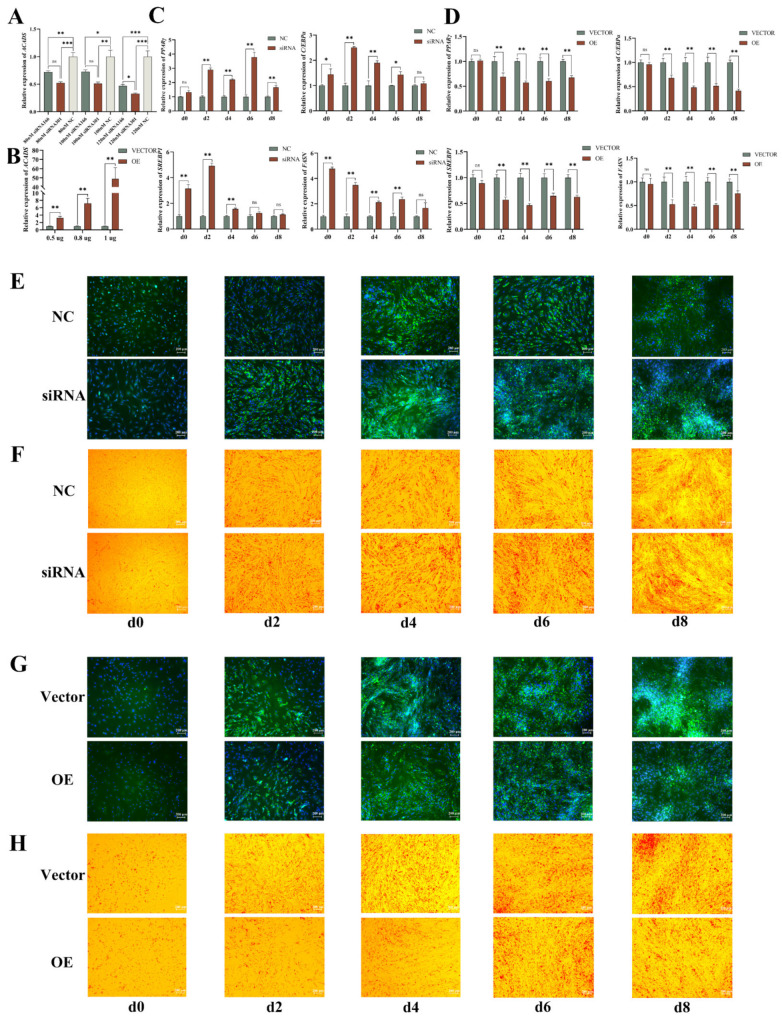
*ACADS* inhibits YIMA lipid accumulation. (**A**) Identification of siRNA interference efficiency and concentration screening of *ACADS* (n = 9). (**B**) Identification of efficiency and concentration screening of *ACADS* overexpression (n = 9). (**C**,**D**) Expression of lipid differentiation marker genes at five time points after *ACADS* interference or overexpression (n = 9). (**E**,**F**) BODIPY staining at five-time points after *ACADS* interference or overexpression. (**G**,**H**) Oil red O staining at five time points after *ACADS* interference or overexpression. * *p* < 0.05, ** *p* < 0.01, *** *p* < 0.001, ns *p* > 0.05. Data are presented as the mean ± SEM.

We successfully inhibited or upregulated *ACADS* expression in cells transfected with siRNA or overexpression vectors (Figure 5A,B) to investigate the impact of *ACADS* on IMF deposition in YIMAs. RT-qPCR analysis revealed that the expression of adipogenic marker genes (*FASN*, *C/EBPα*, *PPARγ*, and *SREBP1*) was significantly upregulated after *ACADS* interference (Figure 5C). Various analyses, including triglyceride (TG) content assays and BODIPY and Oil Red O staining, consistently showed increased lipid deposition in the cells following interference with *ACADS* compared to the NC group (Figure S2A and Figure 5E,F). Moreover, the fatty acid content in YIMAs was significantly reduced after interference with *ACADS* (Figure S2B). Conversely, overexpression of *ACADS* led to results opposite to those observed with interference (Figure 5D,G,H, and Figure S2C). These findings indicate that *ACADS* inhibited the differentiation of YIMAs and promoted the oxidative decomposition of fatty acids, thereby inhibiting IMF deposition in yaks.

### 2.6. ACADS Inhibits YIMA Proliferation

**Figure 6:**
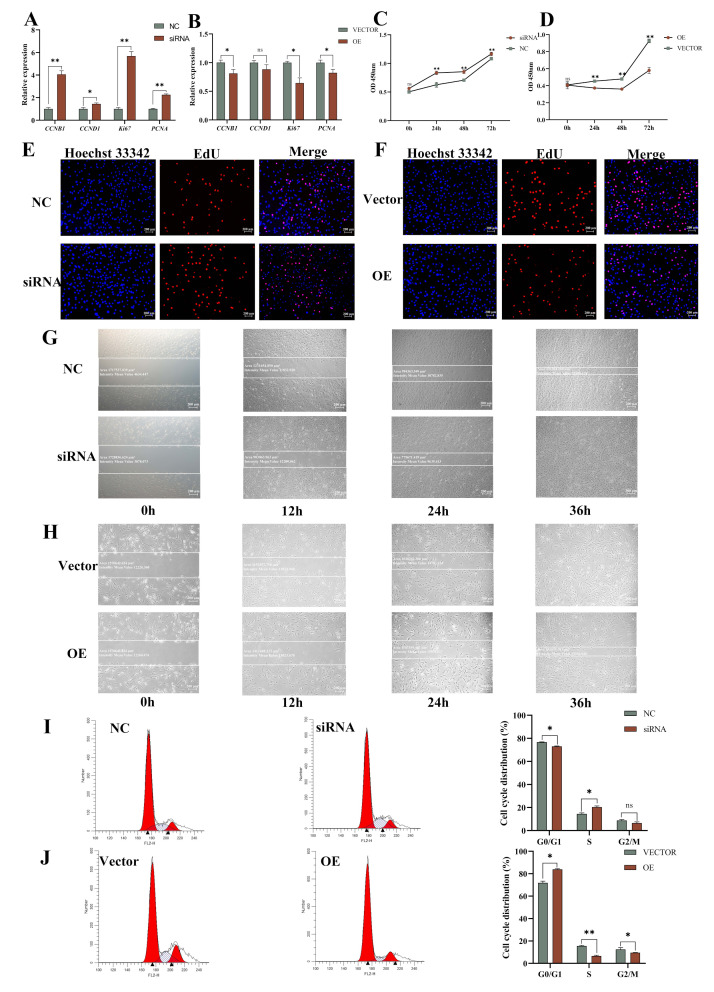
*ACADS* inhibited YIMA proliferation. (**A**,**B**) Expression of proliferation marker genes after interference or overexpression of *ACADS* (n = 9). (**C**,**D**) The OD value of CCK-8 at four time points after interference or overexpression of *ACADS* (n = 6). (**E**,**F**) EdU staining after interference or overexpression of *ACADS*. (**G**,**H**) Scratch test at four time points after interference or overexpression of *ACADS*. (**I**,**J**) Flow cytometry after interference or overexpression of *ACADS*. * *p* < 0.05, ** *p* < 0.01, ns *p* > 0.05. Data are presented as the mean ± SEM.

*ACADS* siRNA and overexpression vectors were transfected into YIMAs at optimal concentrations to verify the role of *ACADS* in the proliferation of YIMAs. As shown in Figure 6A, the cell proliferation marker genes (*Ki67*, *CCNB1*, *CCND1*, and *PCNA*) were significantly upregulated after interference with *ACADS*. These results indicate that interference with *ACADS* promotes the proliferation of YIMAs. CCK-8, EdU, and scratch tests further confirmed these results (Figure 6C,E,G). Conversely, the overexpression of *ACADS* significantly inhibited the proliferation of YIMAs (Figure 6B,D,F,H). The flow cytometry results indicated a significant decrease in the number of cells in the G1 phase and a notable increase in the number of cells in the S phase following interference with *ACADS* compared to the NC group (Figure 6I). In contrast, the cells were mainly blocked in the G0 phase after *ACADS* overexpression (Figure 6J). These results indicate that *ACADS* negatively regulates YIMA proliferation.

### 2.7. ACADS Promotes Mitochondrial Biogenesis and Increases the Mitochondrial Membrane Potential

**Figure 7:**
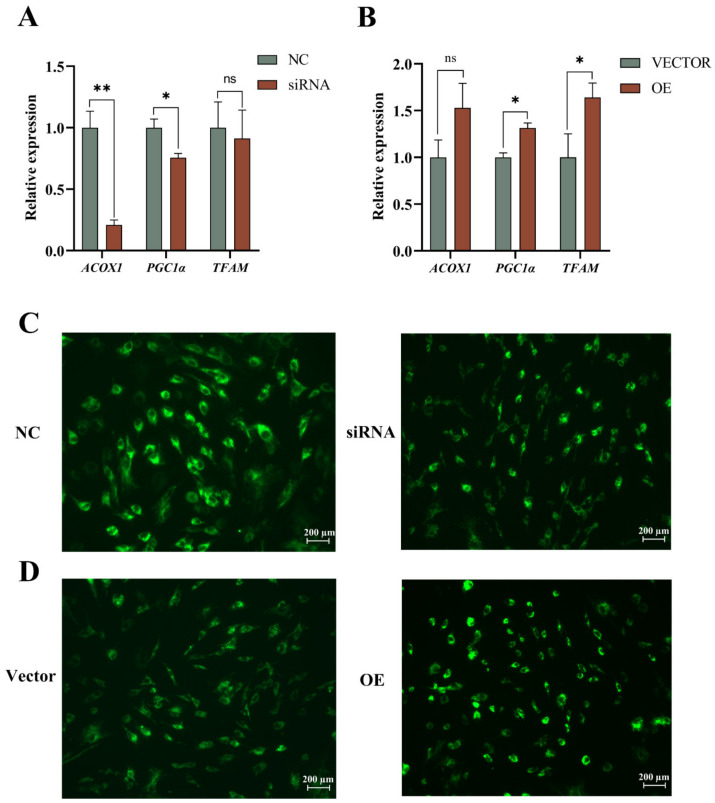
*ACADS* promotes mitochondrial biogenesis and the mitochondrial membrane potential (MMP). (**A**,**B**) Expression of mitochondrial marker genes after interference or overexpression of *ACADS* (n = 9). (**C**,**D**) Rh-123 staining after interference or overexpression of *ACADS*. * *p* < 0.05, ** *p* < 0.01, ns *p* > 0.05. Data are presented as the mean ± SEM.

Mitochondria are the primary sites of fatty acid β-oxidation. In this study, we preliminarily verified the function of *ACADS* in mitochondria. RT-qPCR results showed that interference with *ACADS* inhibited the expression of mitochondrial biosynthesis-related genes *PGC1α*, *TFAM*, and *AOX1* in YIMAs (Figure 7A), while overexpression of *ACADS* had the opposite effect (Figure 7B). Subsequently, Rh123 was used to detect the effect of *ACADS* on the MMP in YIMAs. The results showed that the accumulation of Rh123 decreased after interference with *ACADS* (Figure 7C). However, the opposite results were obtained after the overexpression of *ACADS* (Figure 7D). These results indicate that *ACADS* promotes mitochondrial biosynthesis and increases MMP levels.

### 2.8. ACADS Regulates YIMA Differentiation through the PPARα Signalling Pathway

**Figure 8:**
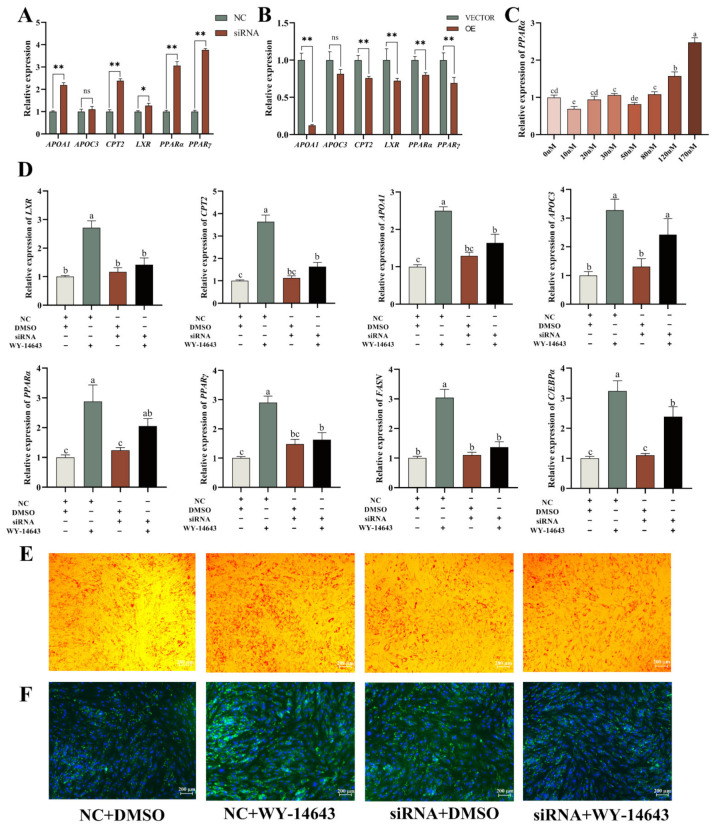
*ACADS* inhibits intramuscular fat (IMF) deposition by regulating the PPARα signalling pathway. (**A**,**B**) Expression of key genes in the PPARα signalling pathway after the interference or overexpression of *ACADS* (n = 9). (**C**) PPARα activator Wy-14643 concentration screening (n = 9). (**D**) Expression of key genes in the PPARα pathway and lipid differentiation marker genes in different treatment control groups (NC, siRNA, Wy-14643, siRNA+Wy-14643), “+” means to add, “−” means not to add, the same lowercase letter indicates that the difference is not significant (*p >* 0.05), the different lowercase letters indicate significant differences (*p <* 0.05). (**E**) Oil red O staining under different treatment control groups. (**F**) BODIPY staining under different treatment control groups. * *p* < 0.05, ** *p* < 0.01, ns *p* > 0.05. Data are presented as the mean ± SEM.

The PPAR signalling pathway is one of the key pathways screened out by omics combined analysis, and previous studies [15] confirmed that PPARα can regulate the expression of *ACADS* (Figure S2D). To verify the relationship between the PPARα signalling pathway and *ACADS*, we determined the expression levels of key genes in the PPARα pathway after the interference or overexpression of *ACADS*. The results showed that *ACADS* could significantly downregulate the expression of key genes in the PPARα signalling pathway (Figure 8A,B), indicating that *ACADS* also exerted a regulatory effect on the PPARα signalling pathway. Wy-14643 is an effective PPARα agonist (Figure 8C), and *ACADS* siRNA and Wy-14643 were co-transfected into YIMAs to verify whether *ACADS* further regulated YIMA lipid deposition by regulating the PPARα signalling pathway. The results showed that after co-transfection of *ACADS* siRNA and Wy-14643, the expression levels of lipid deposition marker genes and PPARα signalling pathway key genes in the Wy-14643 group were significantly higher than those in the siRNA + Wy14643 group (Figure 8D), indicating that siRNA-*ACADS* reversed the promotion of Wy-4643 on PPARα signalling pathway key genes and lipid differentiation marker genes. Lipid droplet staining experiments further verified these results (Figure 8E,F), suggesting that *ACADS* may inhibit YIMA lipid deposition by regulating the expression of key genes in the PPARα signalling pathway.

## 3. Discussion

Yaks inhabit the high-altitude, low-oxygen Qinghai–Tibet Plateau, and the extreme living conditions of the plateau, coupled with free-grazing methods, contribute to the unique characteristics of yak meat [16]. Compared with regular beef from cattle of the same age, yak meat has thicker muscle fibres, lower IMF deposition (2–3%), and lower muscle tenderness, which present certain challenges in terms of processing and meat quality [17]. Thus, a reasonable and effective increase in the IMF content is a key scientific problem that must be resolved to improve the genetic makeup of yaks.

Transcriptome and proteome sequencing was used to screen 256 DEGs and 84 DEPs, respectively. GO and KEGG analyses showed that these DEGs and DEPs were mainly involved in various lipid metabolism processes and thus likely play significant roles in lipid metabolism. A combined analysis of transcriptome and proteome sequencing data was performed to further identify the key genes affecting yak IMF deposition. Five genes (*IGFN1*, *MYL3*, *L2HGDH*, *ACADS*, and *ENSBGRG00000000926*) showed significant differences at both the transcriptome and protein levels, indicating that they may play an important role in the molecular regulation process of regulating yak lipid metabolism. In future research, according to the characteristics of key genes and the biological pathways involved, relevant experiments can be designed to verify the mechanism of their regulation of IMF deposition in yaks so as to provide a reference for elucidating the regulation mechanism of lipid metabolism in yaks and lay a theoretical basis for improving the gene regulation network of IMF deposition in yak.

According to the function of the five genes and differences in their expression at the transcriptome and proteome levels, we selected *ACADS* for subsequent gene function verification. We first cloned the *ACADS* gene of yaks and analysed its tissue and temporal expression profile during cell differentiation. *ACADS* was highly expressed in the liver, which may be related to the involvement of *ACADS* in mitochondrial fatty acid β-oxidation function. Fatty acid β-oxidation is very important for energy metabolism, especially for intermediate metabolism of lipids in the liver [18]. This suggests that *ACADS* may play an important role in regulating liver lipid metabolism. The temporal expression profile revealed that *ACADS* and *PPARγ* expression was the highest on the second day of in vitro differentiation, which was similar to the results of Zhang [19]. The expression trends of *ACADS* and *PPARγ* were consistent during the differentiation process, suggesting that *ACADS* may affect the formation of yak IMF by regulating *PPARγ*. The expression of the *PPARγ* gene after the interference or overexpression of *ACADS* also verified this point. PPI analysis further demonstrated that *ACADS* interacts with several other proteins, including ACOX1, HADHA, HADHB, EHHADH, ACAA2, ACSL1, ACAA1, and ACAA2. These proteins play crucial roles in lipid droplet formation, fatty acid synthesis, and metabolism. For instance, ACSL1 has been found to promote lipid droplet formation in bovine adipocytes [20], ACOX1 inhibits adipogenesis [21], and ACAA1 negatively regulates preadipocyte differentiation in Small-tailed Han sheep [22]. These findings suggest that *ACADS* may be involved in regulating lipid metabolism in yaks.

IMF deposition is a complex biological process involving adipogenic differentiation, preadipocyte proliferation, fat synthesis and decomposition, fatty acid metabolism, and fat transport. At the cellular level, it is mainly manifested as an increase in the number (proliferation) and volume (adipogenic differentiation) of preadipocytes. At the molecular level, it relies on the intricate and precise regulation of fat metabolism-related genes, transcription factors, and epigenetic factors [23]. *PPARγ* is a transcription factor involved in the regulation of adipocyte development and metabolism [24]. *C/EBPα* is also a transcription factor that regulates adipocyte differentiation, mainly by regulating lipid differentiation-related genes to promote lipid deposition [25]. *SREBP-1* is the earliest transcription factor in the process of adipogenesis, and it can promote the expression of *PPARγ*, *ACC*, *FASN*, and other lipid differentiation marker genes [26]. *FASN* is a fatty acid synthase that catalyses fatty acid synthesis and enhances lipid synthesis [27]. In this study, RT-qPCR confirmed that *ACADS* significantly inhibited these genes during the differentiation of yak YIMAs. Lipid droplets are subcellular structures present in eukaryotic cells, and they are characterised by a monolayer phospholipid membrane surrounded by TGs and sterol esters (SEs) [28] and primarily stored in Lipid droplets in the form of neutral lipids [29]. In this study, TG content analyses and BODIPY and Oil red O staining confirmed that *ACADS* inhibited the formation and deposition of TG and Lipid droplets in YIMAs. In addition, RT-qPCR, CCK-8, EdU, and scratch assays showed that *ACADS* significantly inhibited YIMA proliferation. The cell cycle in eukaryotes consists of two stages: the interphase and mitotic (M) phases. Among these, the interphase stage consists of four periods: G0, G1, S, and G2. The G0 phase represents the period when cells exit the cell cycle and cease division; the S phase is an important stage for DNA replication; and the G2 phase is the interval between DNA synthesis and mitosis, during which cells continue to grow [30]. Our results showed that interference with *ACADS* significantly increased the proportion of cells in the S and G2M phases and decreased the proportion of cells in the G0/G1 phase. These results suggest that *ACADS* inhibits the differentiation of YIMAs by regulating the expression of adipogenic marker genes and inhibiting the formation of lipid droplets and TG. It also inhibits the proliferation of YIMAs by regulating the expression of the proliferation marker genes to block the cycle process of YIMAs. Eventually, IMF deposition was inhibited.

Mitochondria play a crucial role in converting carbohydrates, lipids, and proteins into adenosine triphosphate (ATP), thus providing chemical energy to cells. This is achieved through a series of biochemical reactions, including oxidative phosphorylation, the tricarboxylic acid cycle, fatty acid β-oxidation, and pyruvate oxidation [31]. Studies have confirmed that mitochondrial dysfunction can increase lipid droplet content in hepatocytes and induce hepatocyte steatosis [32]. Our results demonstrated that *ACADS* promotes mitochondrial biogenesis and increases the MMP. This suggests that *ACADS* plays a significant role in promoting mitochondrial biosynthesis and metabolic functions. Thus, *ACADS* may promote lipid metabolism by promoting mitochondrial fatty acid β-oxidation, thereby inhibiting IMF deposition.

PPARα is mainly distributed in metabolically active tissues, such as the liver, and controls liver lipid and overall energy balance by regulating three metabolic pathways involved in fatty acid oxidation in the liver [33]. Wy-14643 is a specific agonist of *PPARα*, which is an exogenous ligand [34]. Moreover, fatty acids and their derivatives can be used as endogenous ligands of PPARα [35], such as intermediates produced in the process of fatty acid oxidative metabolism and lipid synthesis [36]. Therefore, *ACADS* may further affect the activation of *PPARα* by regulating the level of intermediate products produced by fatty acid β-oxidation. The experimental results revealed that *PPARα* expression was decreased after *ACADS* overexpression, thus consistent with our hypothesis. As a metabolic sensor, *PPARα* can adjust its activities according to cell energy requirements and lipid availability. In situations where there is an energy deficit, fatty acids are released from peripheral tissues and taken up by the liver. At this time, activated PPARα catalyses the oxidation of fatty acids in the mitochondrial matrix of hepatocytes by regulating key enzymes in lipid metabolism (*ACADS* and ACAA2). This process generates acetyl-CoA and promotes ketogenesis, thus providing fuel to the body [37]. Conversely, in situations of energy excess, *PPARα* can induce adipogenesis [38] and TG metabolism by regulating the expression of a specific group of genes (*FASN* and *SCD1*). Moreover, *PPARα* and *ACADS* may maintain the lipid balance of cells through mutual regulation mechanisms. In this study, *ACADS* demonstrated a significant inhibitory effect on the key genes in the PPARα pathway. Co-transfection of *ACADS* siRNA and Wy-14643 resulted in siRNA-*ACADS* significantly reversing the promoting effect of Wy-14643 on lipid differentiation marker genes and key genes of the PPARα pathway. This suggests that *ACADS* may regulate lipid deposition by modulating the expression of key genes in the PPARα pathway. However, the specific regulatory mechanisms remain to be elucidated.

Although this study verified that *ACADS* has a significant inhibitory effect on the deposition of IMF through a series of experiments, certain limitations must be noted. First, because yaks are mainly distributed within the special growth environment of the Qinghai–Tibet Plateau, significant differences are observed between yaks and ordinary livestock, which leads to discrepancies between the results presented here and those on ordinary cattle. Second, because the sensitivity of proteomics is not sufficiently accurate to detect all proteins, the number of overlapping genes obtained in this study was small. Third, only one internal reference gene was used in qPCR results, which may, to some extent, have affected the results and explanations.

## 4. Materials and Methods

### 4.1. Determination of Intramuscular Fat Content

Twenty-four male Maiwa yaks with similar body weight were selected in Xiaojin County, Sichuan Province, China. After grazing to 4 years old in the plateau pastoral area, they were fattened for 6 months. The ingredients and chemical composition of basic diets fed to yaks were based on previous studies [39]. Then, they (480 + 20 kg) were slaughtered humanely. The LD muscle tissue between the 12th and 13th ribs (right half carcass) of the 24 yaks was taken immediately, and the IMF content was measured via Soxhlet extraction [40]. Three LD tissue samples with the highest IMF content (H-IMF (4.38 ± 0.01) and three LD tissue samples with the lowest IMF content (L-IMF (2.71 ± 0.19)) were selected. The total RNA and total protein of the six samples were extracted and used for transcriptome sequencing (n = 6) and proteome sequencing (n = 6). The part of the study involving animal samples was reviewed and approved by the Institution Animal Care and Use Committee of Southwest Minzu University (Permit number: SMU 202106010), Chengdu, China.

### 4.2. RNA Sequencing (RNA-Seq)

Total RNA was extracted from six LD tissue samples using the Trizol reagent kit (Invitrogen, Carlsbad, CA, USA) following the manufacturer’s protocol. Eukaryotic mRNA was subsequently enriched using Oligo(dT) beads. The enriched mRNA was fragmented into short fragments using a fragmentation buffer and then reverse-transcribed into complementary DNA (cDNA). The purified double-stranded cDNA fragments underwent end repair, the addition of an A base, and ligation to Illumina sequencing adapters (Illumina, San Diego, CA, USA). The ligation reaction was purified using AMPure XP Beads (1.0× concentration) and subjected to polymerase chain reaction (PCR) amplification. Transcriptome sequencing was performed by Gene Denovo Biotechnology Co. (Guangzhou, China).

The screening threshold of DEGs was as follows: |log2 fold change| ≥ 2 and false discovery rate (FDR) ≤ 0.05. The GO and KEGG annotations of the DEGs were performed using the GO (http://www.geneontology.org/, accessed on 10 August 2022) or KEGG (http://www.kegg.jp/kegg/, accessed on 17 August 2022) databases, respectively.

### 4.3. Protein Sequencing

Protein sequencing was conducted by Gene Denovo Biotechnology Co. Total protein from LD samples was extracted through homogenisation. The resulting precipitate was dissolved in lysis buffer (8 M urea, 1% SDS, Protease Inhibitor Cocktail), vortexed, and centrifuged at 15,000× *g* for 5 min at 4 °C. The supernatant was transferred to a new tube, and protein quality was assessed using SDS-PAGE.

Sequence-modified trypsin (Promega, Madison, WI, USA) was added to the protein supernatant and digested overnight at 37 °C. The peptide mixture was desalted by C18 ZipTip, quantified by Pierce™ Quantitative Colorimetric Peptide Assay (23275) and then lyophilized by SpeedVac.

The resultant peptide mixture was labelled with iTRAQ-8Plex Isobaric Mass Tag Labelling Kit (Thermo Fisher Scientific, Waltham, MA, USA) following the manufacturer’s instructions. The labelled peptide samples were then pooled and lyophilized in a vacuum concentrator.

The labelled and freeze-dried peptides were analysed by LCMS/MS equipped with an online nano-spray ion source. Spectronaut was used to analyse the DIA data and the average peak area of the first three MS1 peptides with FDR less than 1.0% was screened for protein quantification. According to the results of protein quantification, proteins with significant changes in abundance between groups were screened out.

The DEPs screening threshold was |log2(fc)| ≥ 1.5, and FDR ≤ 0.05. GO annotation and KEGG analysis were performed on DEPs using their respective databases.

### 4.4. Combined Analysis of Transcriptome and Proteome

The threshold parameters of association analysis were: |log2 (fc)| ≥ 2.0, FDR ≤ 0.05 for DEGs; |log2 (fc)| ≥ 1.5, FDR ≤ 0.05 for DEPs. GO database was selected for functional annotation analysis of DEGs and DEPs. The KEGG pathway database was used to analyse the pathways involved in DEGs and DEPs.

### 4.5. Isolation and Culture of YIMAs

According to previous studies [41], YIMAs were isolated from the LD muscle tissue between the 12th and 13th ribs (right half carcass) of yaks and cultured in an incubator (Semir Fisher Science, Waltham, MA, USA) with 2% CO_2_ at 37 °C. The growth medium was 89% DMEM/F12 (Gibco, Thermo Fisher Scientific) + 10% foetal bovine serum (FBS; Gibco, Thermo Fisher Scientific) + 1% penicillin–streptomycin (P/S; Gibco, Thermo Fisher Scientific). The differentiation medium consisted of a growth medium and 100 μM oleic acid (Merck, Rahway, NJ, USA).

### 4.6. ACADS Overexpression Vector Construction and Cell Transfection

The *ACADS* gene in yaks was cloned using yak cDNA as a template. The CDS of the cloned *ACADS* was subcloned between the *H*ind III and *E*coR I (New England Biolabs, Ipswich, MA, USA) sites of the pCDNA3.1 (+) plasmid using T4 ligase (Takara, San Jose, CA, USA). *ACADS*-specific siRNA was designed and synthesised by Tsingke Biotech (Beijing, China) (Table S2). After cell confluence reached 90%, the *ACADS* overexpression vector and siRNA were transfected (Lipofectamine 3000; Thermo Fisher Scientific) [42].

### 4.7. RNA Extraction, Reverse Transcription, and Fluorescence Quantitative PCR

Total RNA of YIMAs was extracted using TRIzol (Tsingke, Beijing, China). The extracted RNA was reverse-transcribed into cDNA using the PrimeScript RT Master Kit (Takara). Meanwhile, all qPCRs were performed using a SYBR Premix Ex Taq kit (Takara) on a Light Cycler96 Real-Time PCR system (Roche, Munich, Germany). *GAPDH* was used as an internal reference gene. The relative expression levels of the genes were calculated using the 2^−ΔΔCT^ method [43]. All the primers used in this research were designed by Primer5 software (Table S3).

### 4.8. BODIPY and Oil Red O Staining and Triglyceride Analysis

The medium was removed, and the cells were washed with PBS. Next, 4% paraformaldehyde (Biosharp, Hefei, Anhui, China) was added to each well for 30 min to fix the cells. After washing with PBS, Oil red O or BODIPY (Invitrogen) working solutions were added to each well for staining in the dark for 30 min. The cells were then washed with PBS and incubated with DAPI (Solarbio, Beijing, China) staining solution in the dark for 10 min and then washed with PBS. Finally, the cells were photographed under a microscope (Carl Zeiss, Oberkochen, Germany).

Triglyceride (TG) content was measured according to the instructions of the triglyceride kit (Applygen, Beijing, China), and protein content was detected using the BCA assay kit (Invitrogen) to correct the intracellular triglyceride content.

### 4.9. Cell Proliferation Assay

5-Ethynyl-2′-deoxyuridine (EdU) colouring: EdU reagent (Beyotime, Shanghai, China) was added to cells and incubated at 37 °C and 5% CO_2_ for 2 h, then fixed with 4% paraformaldehyde for 15 min at room temperature. Subsequent manipulations were performed according to the manufacturer’s instructions. Samples were photographed using an inverted fluorescence microscope.

Cell counting kit-8 (CCK-8): YIMAs were seeded into 96-well plates and transfected when the cell confluence reached 60%. The medium was replaced with a complete medium containing 10% CCK-8 solution (Vazyme, Nanjing, China) at 0 h, 24 h, 48 h, and 72 h after transfection. The absorbance of each well was measured at 450 nm using a microplate reader (Thermo Fisher Scientific) after incubation for 2 h.

Cell scratch test: the cells were scratched at a constant speed along the central axis of the adherent cells using a sterile gun tip after transfection, and the scratched cells were washed with sterile PBS. Cell proliferation was observed and photographed at 0 h, 12 h, 24 h, and 36 h.

Flow Cytometry: cells were transfected when the cell density reached 80–90%. The cells were collected using trypsin digestion without EDTA (BasalMedia, Shanghai, China) after 48 h, washed with PBS, and fixed with 70% ethanol overnight at 4 °C. PI dye (Solarbio) at a concentration of 50 μg/mL was added and incubated in the dark at 37 °C for 30 min, and the cell cycle was detected using Sysmex Cube 8.0 (Sysmex, Kobe, Japan) platform.

### 4.10. Fatty Acid Content Analysis

The total fatty acids of YIMAs cultured in 60 mm culture dishes were extracted according to previous studies [44]. A HP-88 column (100 m × 0.25 mm × 0.20 μm; Agilent Technologies, Santa Clara, CA, USA) and gas chromatography (7890B; Agilent) were used for analysis. The relative content of each fatty acid component was calculated by referencing the fatty acid standard Supelco 37 Component FAME Mix (Sigma, Burbank, CA, USA). The fatty acid data are expressed as a percentage of each fatty acid to the total fatty acid content.

### 4.11. Measurements of MMP

After treatment, YIMAs were incubated with 5 μg/mL Rhodamine 123 (Rh123; Beyotime) at 37 °C for 45 min. The samples were photographed using a fluorescence microscope.

### 4.12. Statistical Analysis

SPSS 25.0 (IBM Corp, NY, USA) software was used for the statistical analyses. Two groups of samples were tested by independent Student’s *t*-test. One-way analysis of variance (ANOVA) was used for comparison between multiple groups. Duncan’s method was used for multiple comparisons, and values are presented as the mean ± standard error of the mean (SEM). *p* < 0.05 was considered statistically significant and represented by * *p* < 0.05, ** *p* < 0.01, *** *p* < 0.001, and ns = not significant. GraphPad Prism 8 was used to complete data visualisation mapping. The graphical abstract was drawn using Figdraw (https://www.figdraw.com, accessed on 22 October 2023).

## 5. Conclusions

In this study, 256 DEGs and 84 DEPs between the H-IMF and L-IMF groups were identified by transcriptome sequencing and proteomics analysis. Functional verification experiments demonstrated that the *ACADS* gene could inhibit the differentiation and proliferation of YIMAs, promote increases in the MMP, affect the key genes in the PPARα signalling pathway, and ultimately inhibit the deposition of yak IMF. These findings were used to identify potential regulators that may be involved in determining the content of IMF in yaks and provide valuable insights into the role of *ACADS* in molecular breeding for future use in attempts to improve the quality of yak meat.

## Data Availability

The authors confirmed that all data required to evaluate the study’s conclusions are present in the paper. Sequencing data from this study have been submitted to the NCBI database with the registration number PRJNA1014567.

## Supplementary Materials

The following supporting information can be downloaded at https://www.mdpi.com/article/10.3390/ijms25169131/s1.

## Abbreviations

IMF	Intramuscular fat
YIMAs	Yak intramuscular preadipocytes
MMP	Mitochondrial membrane potential
FABP	Fatty acid-binding protein
LPL	Lipoprotein lipase
ATGL	Adipose triglyceride lipase
MAGL	Monoacylglycerol lipase
HSL	Hormone-sensitive lipase
*ACADS*	Acyl-CoA dehydrogenase short-chain
GSC	Glioma sphere-forming cells
MCAD	Medium-chain acyl-CoA dehydrogenase
DEGs	Differentially expressed genes
GO	Gene Ontology
KEGG	Kyoto Encyclopedia of Genes and Genomes
DEPs	Differentially expressed proteins
PCA	Principal component analysis
IGFN1	Immunoglobulin-like and fibronectin type III domain-containing
MYL3	Myosin light chain3
L2HGDH	L-2-hydroxyglutarate dehydrogenase
ATP	Adenosine triphosphate
