# Supplementary material for: Combined Multi-Omics Analysis Reveals the Potential Role of ACADS in Yak Intramuscular Fat Deposition
Source: Int J Mol Sci. 2024 Aug 22;25(16):9131. doi: 10.3390/ijms25169131 (PMC11354380; doi:10.3390/ijms25169131)
Supplement: Supplementary file 1 [file ijms-25-09131-s001.zip › Figure S.pdf]

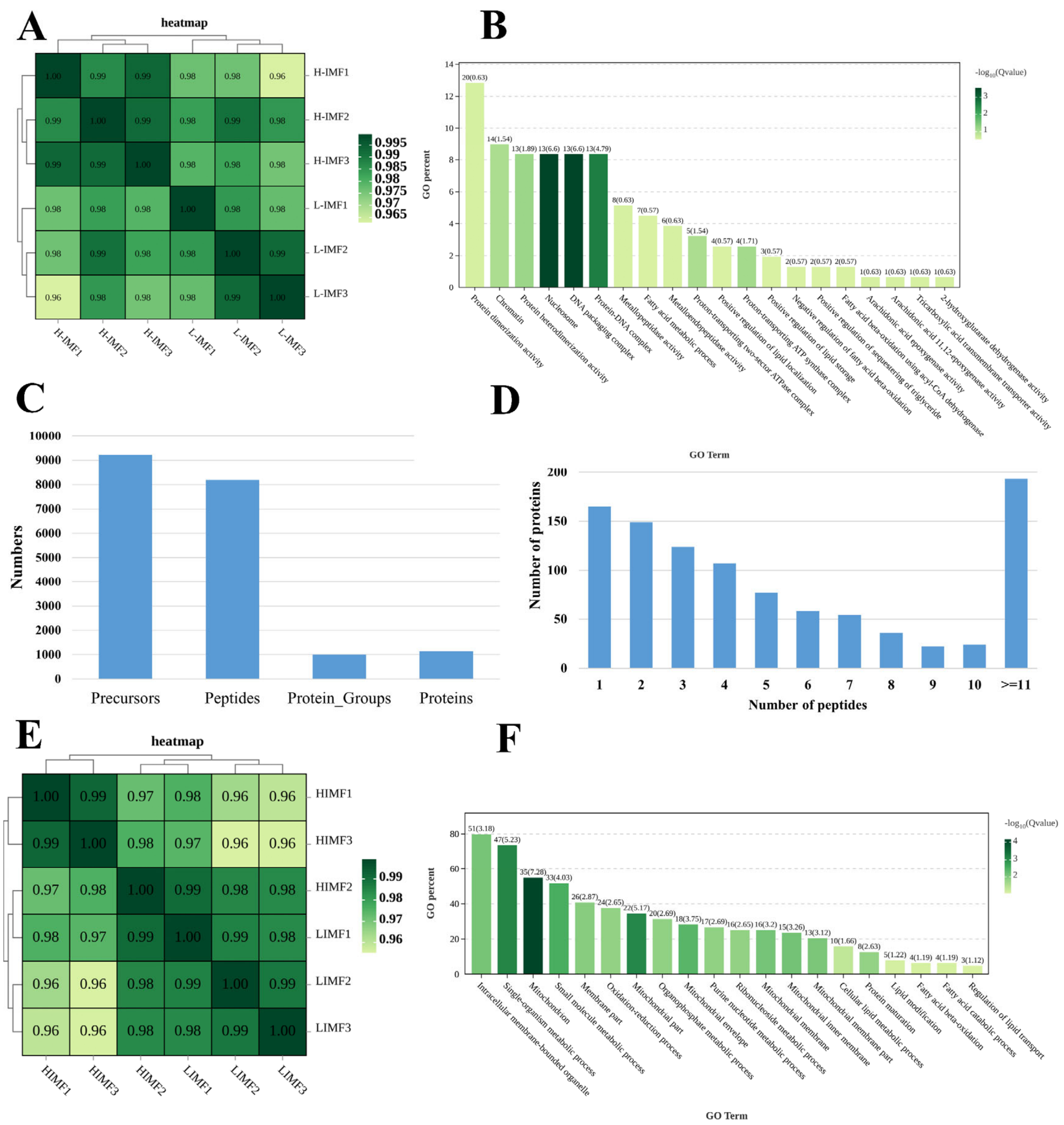

Figure S1. (A) Transcriptome sample correlation heat map; (B) GO enrichment bar figure of DEGs; (C) Basic information statistics for spectra, peptide and protein; (D) Proteome peptide distribution; (E) Proteome sample correlation heat map; (F) GO enrichment bar figure of DEPs.

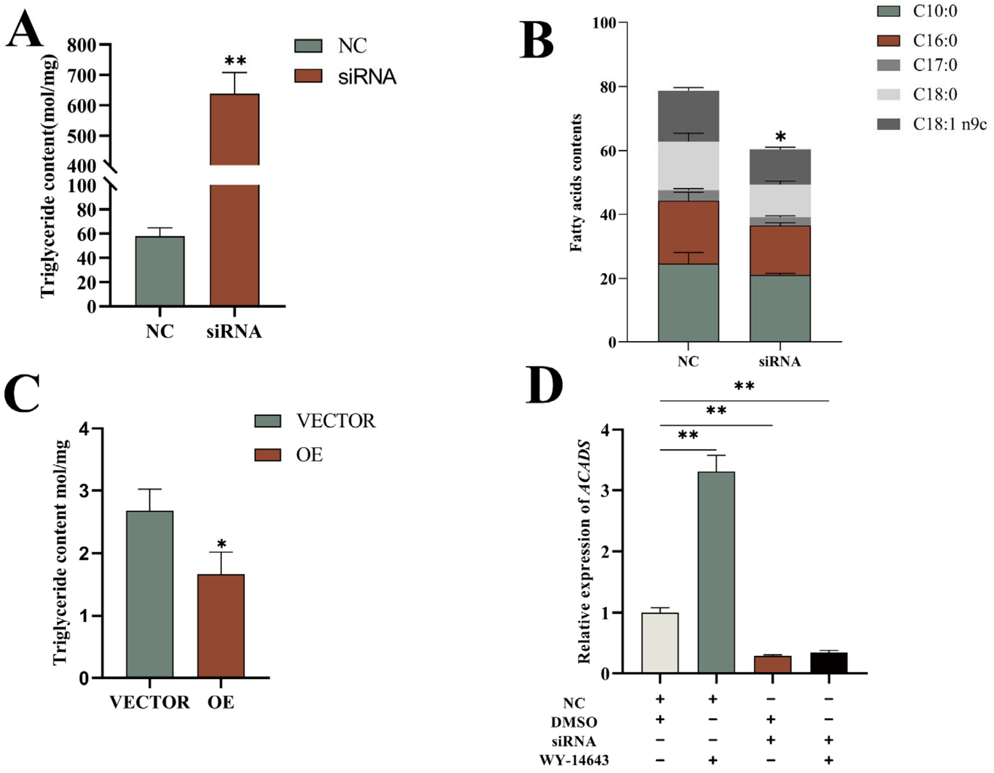

Figure S2. (A) Triglyceride content after interference with *ACADS*; (B) Effect of *ACADS* interference on fatty acid profiles in YIMAs; (C) Effect of *ACADS* overexpression on TG content in YIMAs; (D) The expression of *ACADS* in different treatment control groups.
