# Supplementary figures and images for: Combined Multi-Omics Analysis Reveals the Potential Role of ACADS in Yak Intramuscular Fat Deposition
Source: Int J Mol Sci. 2024 Aug 22;25(16):9131. doi: 10.3390/ijms25169131 (PMC11354380; doi:10.3390/ijms25169131)

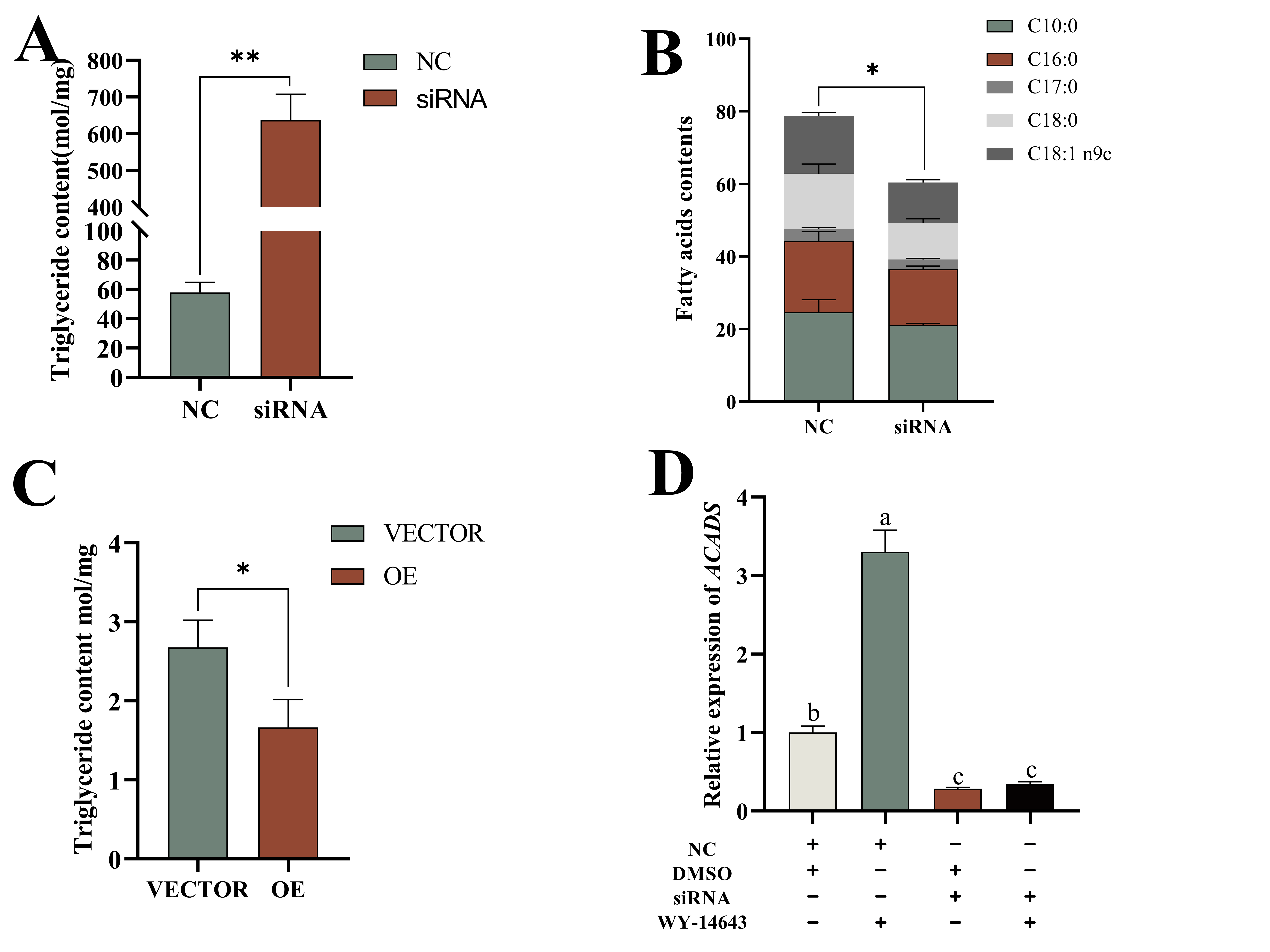

Supplement: Supplementary file 1 [file ijms-25-09131-s001.zip › Figure S2.png]
